# Synthesis and Characterization of Isosorbide-Based Polyurethanes Exhibiting Low Cytotoxicity Towards HaCaT Human Skin Cells

**DOI:** 10.3390/polym10101170

**Published:** 2018-10-20

**Authors:** Barbara S. Gregorí Valdés, Clara S. B. Gomes, Pedro T. Gomes, José R. Ascenso, Hermínio P. Diogo, Lídia M. Gonçalves, Rui Galhano dos Santos, Helena M. Ribeiro, João C. Bordado

**Affiliations:** 1 CERENA, Departamento de Engenharia Química, Instituto Superior Técnico, Universidade de Lisboa, Av. Rovisco Pais, 1049-001 Lisboa, Portugal; barbara.valdes@tecnico.ulisboa.pt (B.S.G.V.); rui.galhano@tecnico.ulisboa.pt (R.G.d.S.); 2 Research Institute for Medicine and Pharmaceutical Science (iMed.ULisboa), Faculty of Pharmacy, Universidade de Lisboa, Av. Prof. Gama Pinto, 1649-003 Lisboa, Portugal; lgoncalves@ff.ulisboa.pt (L.M.G.); hribeiro@campus.ul.pt (H.M.R.); 3 Centro de Química Estrutural, Departamento de Engenharia Química, Instituto Superior Técnico, Universidade de Lisboa, Av. Rovisco Pais, 1049-001 Lisboa, Portugal; clara.gomes@tecnico.ulisboa.pt (C.S.B.G.); hdiogo@tecnico.ulisboa.pt (H.P.D.)

**Keywords:** biocompatibility, GPC/SEC, keratinocyte cells, NMR, polyurethane, renewable sources

## Abstract

The synthesis of four samples of new polyurethanes was evaluated by changing the ratio of the diol monomers used, poly(propylene glycol) (PPG) and D-isosorbide, in the presence of aliphatic isocyanates such as the isophorone diisocyanate (IPDI) and 4,4′-methylenebis(cyclohexyl isocyanate) (HMDI). The thermal properties of the four polymers obtained were determined by DSC, exhibiting *T*_g_ values in the range 55–70 °C, and their molecular structure characterized by FTIR, ^1^H, and ^13^C NMR spectroscopies. The diffusion coefficients of these polymers in solution were measured by the Pulse Gradient Spin Echo (PGSE) NMR method, enabling the calculation of the corresponding hydrodynamic radii in diluted solution (1.62–2.65 nm). The molecular weights were determined by GPC/SEC and compared with the values determined by a quantitative ^13^C NMR analysis. Finally, the biocompatibility of the polyurethanes was assessed using the HaCaT keratinocyte cell line by the MTT reduction assay method showing values superior to 70% cell viability.

## 1. Introduction

Polyurethanes (PUs) are extremely versatile polymers due to their easy structural tunability and to their elastomeric and thermoplastic behavior. Their syntheses from the combination of new monomers are being extensively studied by several groups [1,2,3,4,5,6].

PU materials are usually composed of two types of phases, in which *hard* glassy phase-enriched domains are dispersed in a matrix of *soft* rubbery segments. These *hard* segments contribute to the modulus, mechanical strength and elevated temperature properties owing to the strong intermolecular interactions such as hydrogen bonding among the urethane groups, whereas the *soft* segments afford elasticity and low-temperature mechanical properties [2]. Moreover, the properties of PUs are remarkably affected by the content, type, and molecular weight of the soft segments. Therefore, the choice of monomers used to synthesize PUs is dependent on the final applications of the materials [7]. For example, monomers offering excellent durability are selected for polymers to be used for prostheses [8], those increasing the compatibility of PU with tissues are applied for pacemakers [9], and monomers offering thermal stability are carefully chosen for catheters that require sterilization processes [7,10].

**Figure 1:**
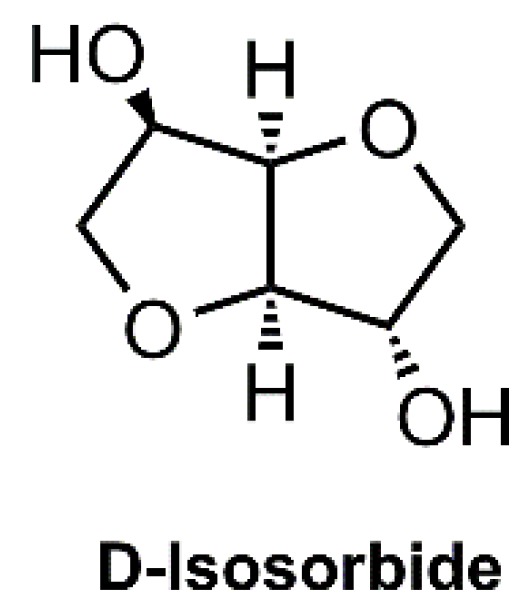
The molecular structure of D-isosorbide.

We have been interested in the synthesis of linear polyurethanes from carbohydrates suitably functionalized for applications in the pharmaceutical industry, namely, for the use in nail lacquer formulations, as they are being considered as a solution to deliver relevant drugs to infected nails [11]. However, the currently available polymer excipients present some disadvantages for the formulation of vehicles for an efficient drug delivery using therapeutic nail lacquers. Isosorbide is a monomer already employed in the synthesis of biocompatible polymers [3,7,12,13]. This glycol (Figure 1) is a chiral and quite thermostable diol, which raises the *T*_g_ of the corresponding polymers, and can be obtained by the reduction of glucose followed by dehydration [14]. On the other hand, the poly(propylene glycol) (PPG) is a biocompatible polyether that has been extensively investigated for application as a biomedical material conferring elastomeric properties to polyurethanes [15,16,17]. This monomer was then chosen as a second glycol in the outlined synthesis. To avoid the use of monomers that probably yield toxic compounds, the isophorone diisocyanate (IPDI) and 4,4′-methylenebis(cyclohexyl isocyanate) (HMDI) were envisaged as options to access nontoxic formulations since those isocyanates have already been used in the synthesis of several biocompatible polyurethanes [16,18,19].

The present work describes the synthesis of new polyurethanes from D-isosorbide by step-growth polymerization involving diisocyanates, such as IPDI or HDMI, and PPG as soft segments, and the characterization of their chemical structures, molecular weights, and thermal properties by Fourier-Transform Infrared (FTIR) and ^1^H and ^13^C Nuclear Magnetic Resonance spectroscopies (NMR), Gel Permeation Chromatography/Size-Exclusion Chromatography (GPC/SEC), and Differential Scanning Calorimetry (DSC). The novelty of this work consists in the synthesis and characterization of new polyurethanes derived from unprecedented combinations of aliphatic monomers (PPG-HMDI-Isosorbide and PPG-IPDI-Isosorbide), which are biocompatible with keratinocyte cells and can participate as drug delivery supports in the formulation of nail lacquers used in the treatment of onychomycosis. In fact, to the best of our knowledge, there are no reports on the application of D-isosorbide-based polyurethanes in therapeutic nail lacquers.

## 2. Experimental Section

### 2.1. Materials

The D-isosorbide was provided by Acrös (Geel, Belgium), HMDI by Bayer Material Science (Leverkusen, Germany), and IPDI and 2,2′-dimorpholinyldiethylether (DMDEE) by Sigma-Aldrich (Ludwigshafen, Germany). PPG (VORANOL 1010L, average molecular weight 1000 g mol^−1^, OH number range (Phthalic Anhydride-Pyridine Solution) 106–114 mg KOH g^−1^) was supplied by Dow Chemical (Midland, MI, USA). Anhydrous ethanol was purchased from Carlo Erba (Barcelona, Spain). DMSO-*d*_6_, CDCl_3_, and tetramethylsilane (TMS) (99.9% purity) used in the NMR determinations were acquired from Sigma-Aldrich. The HPLC grade tetrahydrofuran (THF) and the polystyrene (PS) standards used in GPC/SEC were obtained from Aldrich and TSK Tosoh Co. (Tokyo, Japan), respectively.

### 2.2. Polymer Synthesis

The polyurethanes reported in the present work were synthesized in two steps. The first one involved the quasi-prepolymerization method by reacting the IPDI or HMDI monomers and PPG for ca. 3 h, at 80 °C. Subsequently, the second step comprised the reaction of the previously afforded quasi-prepolymers with D-isosorbide diol monomer for 1 h, at 80 °C.

#### 2.2.1. Synthesis of Quasi-Prepolymers

Three polyurethane quasi-prepolymers were prepared by reacting IPDI or HMDI and PPG in a 250 mL glass flask, under a dry nitrogen atmosphere, to prevent and avoid the presence of moisture and the subsequent formation of urea bonds during the synthesis. The reaction took place with the dropwise addition of PPG to the quasi-prepolymer, under mechanical stirring (400 rpm), at 80 °C and in the presence of 0.5 mL of the catalyst DMDEE. The reactions were practically completed in 3 h, as determined by the isocyanate (-NCO) groups content existing in the reaction mixtures (determined by back titration with an excess of *N*,*N*-dibutylamine with standard HCl [20]). Viscometry measurements of the reaction mixtures by cone-plate rheometry were also used to evaluate the course of the reaction [21] (using a shear stress-controlled ICI Cone and Plate rheometer—London, LTD—at 25 °C and 20 Hz, using parallel plates, with an upper plate diameter of 20 mm and a gap of 0.4 mm). The molar ratios of the monomers used in each synthesized pre-polymer are presented in Table 1.

#### 2.2.2. Syntheses of Polyurethanes PU1–PU4

The syntheses of the four polyurethanes **PU1**–**PU4** were carried out in a 250 mL reactor by the addition of an appropriate amount of D-isosorbide to the IPDI/PPG or HMDI/PPG polyurethane quasi-prepolymers obtained previously (see Section 2.2.1), according to the predetermined molar and mass ratios of monomers and prepolymer, which are presented in Table 2. For comparison, the hard segment content, as obtained by the NMR for each **PU**, is also presented (see below in Table 5). The reactions took place in 1 h at 80 °C under a dry nitrogen atmosphere, in quantitative yields.

### 2.3. FTIR Characterization

The quasi-prepolymers’ FTIR spectra were acquired with a Nexus Thermo Nicolet spectrometer equipped with attenuated total reflectance (ATR) device for the quasi-prepolymers. The solid samples of polyurethane (approximately 1 mg) were finely powdered and dispersed in a KBr matrix (200 mg). For each polymer, a pellet was then formed by compressing the sample at 784 MPa. The FTIR spectra were obtained at room temperature, in the range of 4000 to 600 cm^−1^, after 128 scans with a resolution of 4 cm^−1^.

### 2.4. NMR Spectroscopy Characterization

^1^H and ^13^C NMR spectra were obtained using a Bruker AVANCE III 500 MHz spectrometer (Bruker Corporation, Billerica, MA, USA) equipped with a 5 mm BBO probe. To prepare each of the polyurethanes samples, 20–53 mg of the polymers were dissolved in 0.6 mL of DMSO-*d*_6._ Quantitative ^13^C NMR spectra were obtained under inverse gated decoupling with a delay of 9 s and an 80-degree pulse. Two-dimensional COSY, HSQC, and HMBC spectra were obtained with Bruker standard sequences. All the spectra were run at room temperature employing TMS as an internal standard.

### 2.5. Measurement of the Diffusion Coefficients by NMR Spectroscopy

The Pulse Gradient Spin Echo (PGSE) method is a relatively simple technique of measuring diffusion coefficients in solutions. It combines NMR spin echoes with pulsed-field gradients of variable strength. To determine the diffusion coefficient of a molecule in a solution, a series of ^1^H spectra is obtained in which the strength of the gradients is increased and the attenuation of the intensity of the proton peaks due to diffusion is monitored [22]. In our study, the diffusion coefficient (*D*) of each polymer was determined at five different concentrations. For this purpose, a series of five solutions was prepared with concentrations 0.03%, 0.07%, 0.15%, 0.3%, and 1% (*w*/*v*) in DMSO-*d*_6_. The diffusion coefficients of the polymers at infinite diffusion (*D*_0_) were obtained from plots of *D* versus concentration (Tables S1–S4 and Figure S12 in the Supplementary Material). The *D* values were measured using the PGSE method in an NMR Bruker AVANCE III 500 MHz spectrometer with a 3-mm BBO probe and a z-gradient shielded coil. This combination gives a maximum possible gradient of 0.56 T m^−1^. A bipolar stimulated echo sequence (STE) with smoothed square gradients and WATERGATE solvent suppression were used [23]. The signal intensity was monitored as a function of the square of the gradient amplitude and the resulting self-diffusion coefficients *D* were calculated according to the echo attenuation equation for STE:

*I* = *I*_0_ exp[−*D* (γδg)^2^ (∆ − δ/3)] (1)

where *I*_0_ is the intensity in the absence of gradient pulses, δ is the duration of the applied gradient, γ is the gyromagnetic ratio of the nucleus, and Δ is the diffusion time. For each polymer, the areas of three or four single proton peaks were used in the fittings and the average *D* value was taken. For more details see the Supplementary Materials. 

The solutions of the polymers were transferred to 3 mm NMR tubes to a total volume of 0.3 mL. To guarantee reproducibility of the results this volume was kept constant in all the samples. The temperature was controlled at 30 °C by a BCU05 Bruker unit with an air flow of 521 Lh^−1^.

### 2.6. Gel Permeation Chromatography/Size-Exclusion Chromatography (GPC/SEC) Characterization

The analyses were made in an HPLC Waters chromatograph containing a Waters 515 isocratic pump and a Waters 2414 refractive index detector. In this apparatus, the oven was stabilized at 35 °C and the elution of samples was carried out through two PolyPore columns (Agilent, Santa Clara, CA, USA), protected by a PolyPore Guard column (Agilent). The software Empower^®^ performed the acquisition and data processing.

THF was used as the eluent, at a flow rate of 1.0 mL min^−1^. Before use, the solvent was filtered through 0.45 μm of PTFE membranes Fluoropore (Merck Millipore, Burlington, MA, USA) and degassed in an ultrasound bath for 45 min. The oligomer/polymer samples were also filtered across 0.20 μm PTFE filters Durapore (Merck Millipore). The molecular weights were calibrated relative to polystyrene standards (TSK Tosoh Co.). As a result, it should be taken into account that small deviations could have occurred when the present polymer samples were analyzed.

### 2.7. Thermal Behavior Characterization by Differential Scanning Calorimetry (DSC)

The characterization of the thermal behavior of the polymer samples was performed with a 2920 MDSC system from TA Instruments Inc. (New Castle, DE, USA), equipped with a refrigerated cooling accessory (LNCA), which provided automatic and continuous programmed sample cooling down to 123 K.

The temperature scale of the instrument was calibrated with five standard compounds and the heat flow scale was calibrated with indium and tin. Further details regarding the calibration process can be found in the literature [24]. The samples were accurately weighed (±0.1 µg) in aluminum pans on a Mettler UMT2 ultra-micro balance in the air. All the measurements were performed under dry high purity helium gas (Air Liquide N55 – Alphagaz 1, Paris, France), at a flow rate of 30 mL min^−1^.

The protocol used in the DSC measurements consisted of two consecutive identical thermal cycles: samples were cooled to −110 °C and then heated until 230 °C, at 10 K min^−1^.

### 2.8. In Vitro Cytotoxicity Assay

The polyurethanes were completely dissolved in ethanol under stirring (200 rpm), at a concentration of 250 mg mL^−1^. Later, this solution was spread on one side of a 10 mm diameter glass coverslip and dried for 30 min.

The biocompatibility of the polymer was evaluated in vitro by direct contact with cells, following the ISO 10993-5:2009 recommendation guidelines [25]. The procedure used was previously published in Reference [11]. Briefly, to each polyurethane glass coverslip was added 1.25 × 10^5^ cells HaCaT (Cultured Human Keratinocyte) cell suspension in the culture medium, in sterile 24-well plates. The plates were incubated for 72 h in a humidified atmosphere of 5% CO_2_ at 37 °C without refreshing the culture medium. For cell proliferation quantification, the general cell viability endpoint MTT reduction was used (MTT = 3-(4,5-dimethylthiazol-2-yl)-2,5-diphenyltetrazolium bromide).

The data are expressed as the mean and the respective standard deviation (mean ± SD) of 6 experiments. The statistical evaluation of data was performed using one-way analysis of variance (ANOVA). The Tukey–Kramer multiple comparison tests (GraphPad PRISM 5 software, GraphPad Software Inc., La Jolla, CA, USA) were used to compare the significance of the difference between the groups; a *p* < 0.05 was accepted as statistically significant.

## 3. Results and Discussion

### 3.1. Syntheses of the PUs

A family of aliphatic biocompatible PUs, based on IPDI or HDMI, D-isosorbide and PPG, were synthesized in order to convey the active principle. The PUs were designed to be composed of various ratios of soft segments (PPG based blocks) and hard segments (D-isosorbide based blocks). 

**Figure 2:**
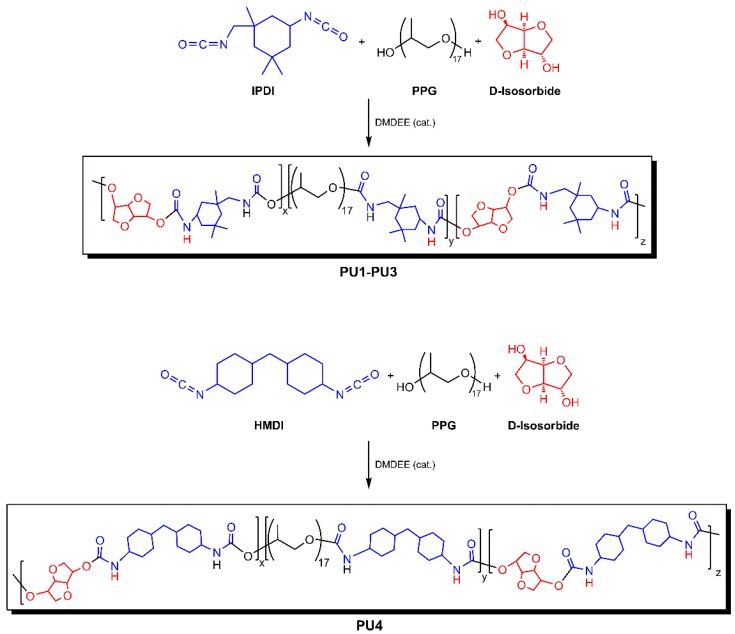
The synthesis of polyurethanes **PU1**–**PU4**.

A two-stage step-growth polymerization method was employed. In an early step, the pre-polymerization of an excess of a diisocyanate monomer (IPDI or HMDI) with the PPG diol, catalyzed by DMDEE, was carried out by stirring the reaction mixture for 3 h, at 80 °C. Then, a complementary step consisted in the addition of a pre-defined amount of the D-isosorbide diol monomer to the previous pre-polymer reaction mixtures (catalyst included), at the abovementioned temperature, for an additional hour. The polyurethanes **PU1**–**PU3** were obtained from the reaction of IPDI, PPG, and D-isosorbide, according to the ratios listed in Table 2, whereas **PU4** was obtained from the reaction of HMDI, PPG, and D-isosorbide (Figure 2). The four PU products obtained in these reactions were characterized by FTIR, NMR, and GPC/SEC without further purification.

### 3.2. FTIR of the PUs

**Figure 3:**
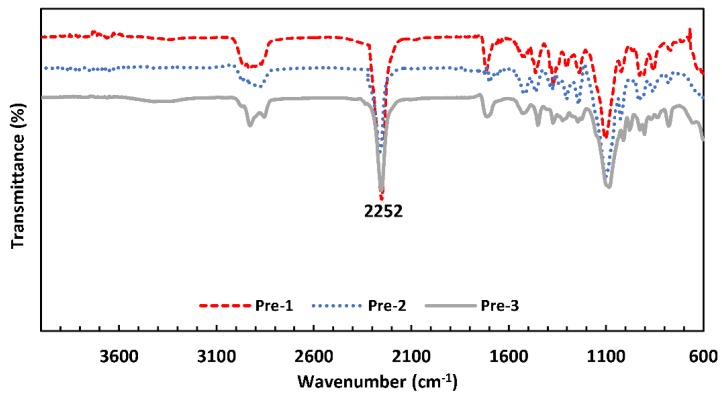
The FTIR spectra of the three polyurethane quasi-prepolymers.

The FTIR spectra of the three quasi-prepolymers, which were synthesized according to the monomer molar ratios defined in Table 1, are presented in Figure 3. In all the quasi-prepolymers, a band at 2252 cm^−1^ is observed, indicating the presence of isocyanate groups (–NCO) corresponding to the monomers IPDI and HMDI, which is in agreement with the reports of the literature (2270 to 2250 cm^−1^ for the N=C=O stretching vibration) [26,27,28]. The spectra of the quasi-prepolymers also showed bands at 2927 cm^−1^, corresponding to –CH_2_- groups, and the peak of the ether group at 1527 cm^−1^. 

These bands correspond to the PPG segments (pure PPG presents a –CH_2_– band between 2970–2850 cm^−1^ and another one corresponding to the ether group at 1526 cm^−1^ [29]). The band corresponding to the carbonyl groups is observed between 1677–1708 cm^−1^, which is indicative of the condensation reaction between the –OH group of PPG and the –NCO groups of HMDI and IPDI [30,31,32].

**Figure 4:**
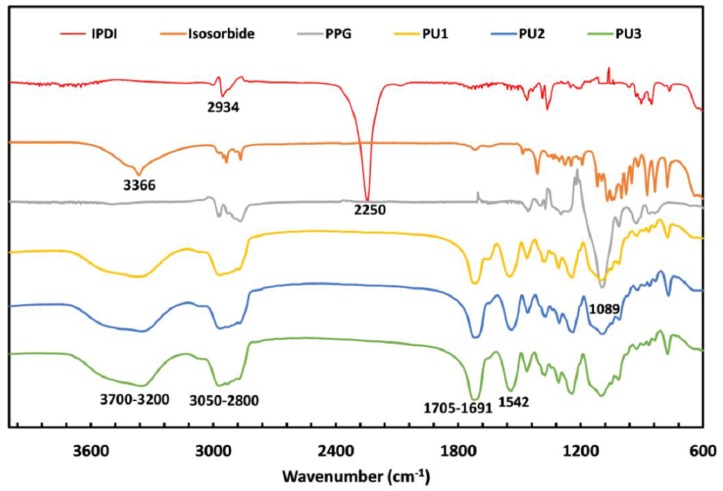
The FTIR spectra of the monomers Isosorbide, PPG, IPDI, and of the polyurethanes **PU1**‒**PU3**.

The FTIR spectra of the resulting IPDI based polyurethanes **PU1**–**PU3** and the corresponding monomers are shown in Figure 4. The characteristic band of the isosorbide monomer –OH groups is observed at 3366 cm^−1^, whereas the band at 2973 cm^−1^ corresponds to the isosorbide methylene group (–CH_2_–) [33]*.* In the PUs spectra, a broad signal between 3700 and 3200 cm^−1^ remains. This signal likely corresponds to the superimposition of –NH– stretching vibrations of the urethane groups of the PUs hard segments as well as to terminal isosorbide –OH groups and terminal urea –NH_2_ groups (the latter resulting from the hydrolysis of the PUs’ former –NCO urethane terminal groups upon exposure to air after the reaction). The –CH_2_– bands, identified in isosorbide and PPG, are detected in the spectra of the PUs between 3050 and 2800 cm^−1^. The hard segments carbonyl bands can be observed between 1705–1691 cm^−1^ [30,34]. The disappearance of –N=C=O stretching vibration, around 2250 cm^−1^, in the spectra of the synthesized polyurethanes suggests that there are no unreacted isocyanate groups [19], i.e., all the isocyanate groups reacted with the monomers –OH groups (and the remaining terminal ones with H_2_O from moisture) [35].

On the other hand, the –NH– bending bands of the synthesized PUs were identified at 1542 cm^−1^ (Figure 4) [27]. These bands were observed in the reaction between polycaprolactone and 2-isocyanate ethylmethacrylate [36]. The –NH– bending of the urethane group was also detected by da Silva et al. at the same wavenumber [37]. Furthermore, C–O–C stretching was observed at 1089 cm^−1^ [34].

**Figure 5:**
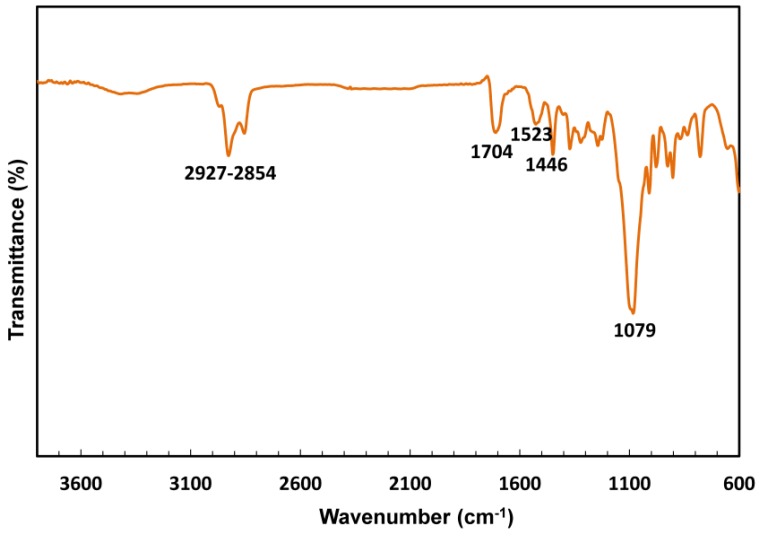
The FTIR spectrum of the polyurethane **PU4**.

The FTIR spectrum of the HMDI based polyurethane **PU4** is depicted in Figure 5. In this spectrum, the –CH_2_– stretching bands (from PPG) are observed between 2927 and 2854 cm^−1^, the band of the urethane carbonyl being detected at 1704 cm^−1^. The C–N stretching bands, combined with those of the bending of N–H in the plane, are typically observed at 1523 cm^−1^ and 1446 cm^−1^, demonstrating the occurrence of the reaction between the hydroxyl group and the isocyanate [38]. The C–O–C characteristic band was identified at 1079 cm^−1^.

### 3.3. NMR of the PUs

**Figure 6:**
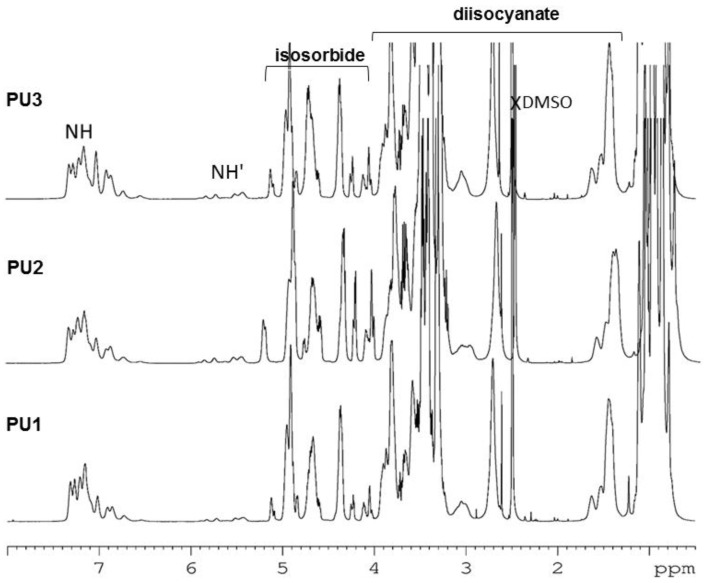
The ^1^H NMR spectra of **PU1**, **PU2**, and **PU3** in DMSO-*d*_6_. Protons of the terminal groups are primed.

The ^1^H NMR spectra of polyurethanes **PU1**, **PU2**, and **PU**3 are shown in Figure 6.

**Figure 7:**
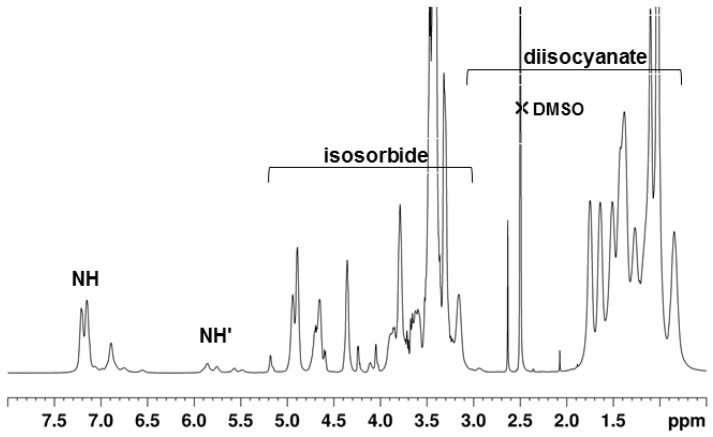
The ^1^H NMR spectrum of **PU4** in DMSO-*d*_6_. Protons of the terminal groups are primed.

The presence of amide protons near 7.0 ppm of the urethane groups –OCONH– indicates that the reaction between the isosorbide hydroxyl groups and the pre-polymer isocyanate groups has occurred. In the research published by Besse et al., the characteristic –NH– of the urethane group was identified at 7.1 ppm as a result of the synthesis of polyhydroxyurethanes by the reaction of isosorbide dicyclocarbonate with four commercial diamines [3]. Other authors mentioned that the proton from the –OCONH– group, obtained from the reaction between IPDI and a polyester, can also be observed at 6.8, 6.82, and 7.2 ppm [39]. The literature data shows data in the polyurethanes resulting from the reaction between isosorbide, hexamethylene diiosocyanate (HDI), and poly(tetramethylene glycol) (PTMG); the –NH– peak is described at 8.07 ppm [7]. The ^1^H NMR spectra of the in-chain isosorbide units, peaks from 4.0 to 5.2 ppm; of the IPDI units, peaks from 2.6–4.0 ppm; and of the IPDI methyl groups, ca. 1.0 ppm, were assigned on the basis of the existing literature of pre-polymers made from the reaction of PPG with IPDI monomers [17]. The assignment of the isosorbide and IPDI resonances in the spectra of **PU1**–**PU3** were further confirmed by means of 2D NMR experiments (COSY, HSQC, and HMBC). Some of these spectra are shown in Figures S1 and S3–S7 of the Supplementary Materials. The peaks of the ^1^H NMR spectra of the PPG soft-segment are observed as multiple resonances between 3.2 and 4.0 ppm for both the CH and CH_2_ protons, and in the range 1.0–1.2 ppm for the methyl groups. The ^1^H NMR spectrum of **PU4** is presented in Figure 7. The peaks ca. 7.2 ppm in Figure 7 corresponds to the NH of urethane groups. The signals at 4.0–5.2 ppm are a piece of evidence for the presence of isosorbide groups in the structure of polyurethane [18]. The strong peaks centered at 1.2 and 3.4 ppm belong to the CH_3_ and CH+CH_2_ protons of the PPG segment, respectively. The peaks characteristic of HMDI units are identified at 0.75–3.6 ppm [31,40]. The assignment of the spectra of the isosorbide and HMDI units were further confirmed by means of 2D NMR experiments (HSQC and HMBC). They are shown in Figures S8–S11 of the Supplementary Materials. The unreacted hydroxyl in *exo* position of the isosorbide end group appears as a minor resonance at ca. 5.1 ppm and is characteristic of polyurethanes with isosorbide as a chain extender [19].

**Figure 8:**
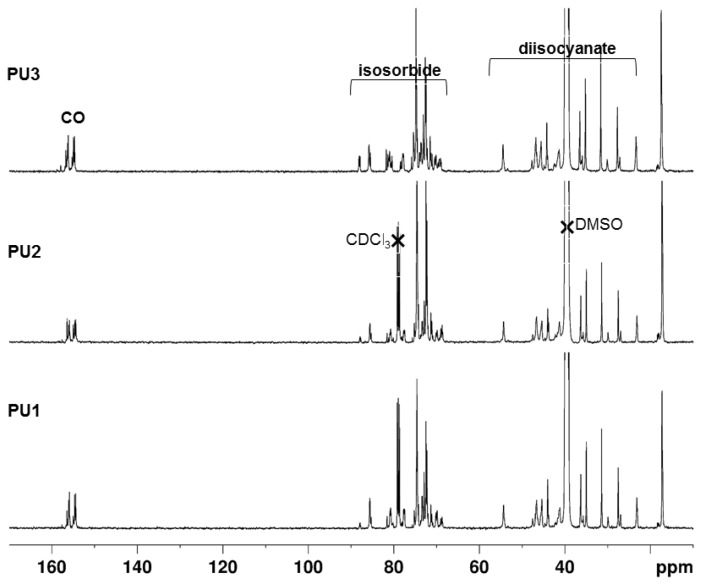
The ^13^C{^1^H} NMR spectra of **PU1**, **PU2**, and **PU3** in DMSO-*d*_6_. Traces of CDCl_3_ are seen in the spectra of **PU1** and **PU2**.

The assignment of the ^13^C NMR spectra of polyurethanes **PU1**, **PU2**, and **PU3** presented in Figure 8 was complicated owing to the fact that monomer IPDI is available as a mixture of the *cis* and *trans* isomers (3:1), and we had to use HSQC and HMBC spectra to fully assign the in-chain carbons (see Figures S2 and S4–S7 of the Supplementary Materials). The peaks from the isosorbide units are observed between 65 and 90 ppm, while those of IPDI appear at a higher field from 20 to 60 ppm. The carbonyl resonances appear between 153–157 ppm while the strong peaks centered at 72.4 and 74.6 ppm correspond to the methine and methylenic carbons of the PPG segments, respectively. The methyl peaks appear at around 17.2 ppm. The experimental ^13^C chemical shifts agree with those referenced in the literature [17] for pre-polymers made of PPG and IPDI monomers.

No peaks of the primary and secondary isocyanate groups of the IPDI terminal groups or monomers appear at 121.7 and 122.6 ppm in the spectra [41], which indicates that all the IPDI monomers/units have reacted with the -OH groups of the diol monomers, in accordance with the FTIR results.

**Figure 9:**
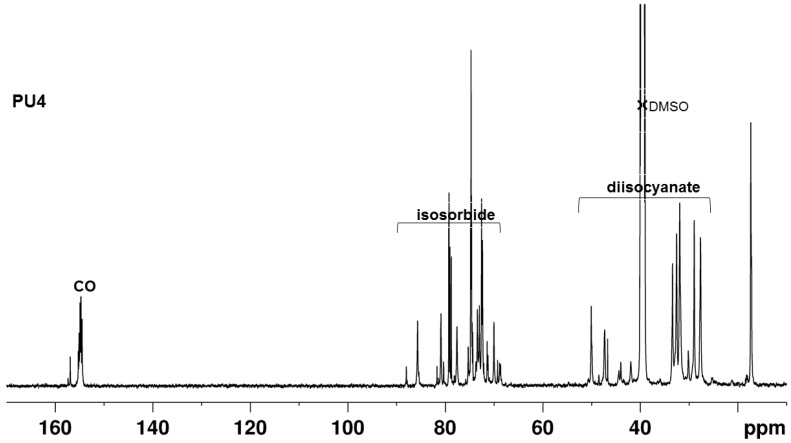
The ^13^C{^1^H} NMR spectrum of the **PU4** in DMSO-*d*_6_.

The ^13^C{^1^H} NMR spectrum of **PU4** presented in Figure 9 is similar to those of the previous PUs, except that the higher field region is occupied by the peaks of the HDMI monomers. HSQC and HMBC spectra were used to fully assign the in-chain carbons of **PU4** (see Figures S9–S11 of the Supplementary Materials). This similarity is found in the characterization of polyether urethane urea obtained by the reaction between HMDI and polyethylene glycol and ethylenediamine [42].

The disappearance of the ^13^C NMR peak at 124 ppm, characteristic of the isocyanate group, indicates that all the diisocyanate groups have fully reacted with the diol monomers [31]. This result was also confirmed by the FTIR analysis.

### 3.4. Determination of Hydrodynamic Radii of the PUs

The determination of the diffusion coefficients of the PUs of this work by the Pulse Gradient Spin Echo (PGSE) NMR method, enabled the calculation of the corresponding hydrodynamic radii (*r*_h_) using the Stokes-Einstein equation for diluted solutions of large molecules [43]:

(2) $$D_{0}=\frac{k_{B}T}{6\pi \eta r_{h}}$$

where *k*_B_ is the Boltzmann’s constant, *T* is the temperature, η is the viscosity of the solution (N s m^−2^ = kg s^−1^ m^−1^) and *r*_h_ is the hydrodynamic radius (m). The viscosity of DMSO-*d*_6_ at 30 °C, η = 1.951 × 10^−3^ Pa.s, was estimated from that of the non-deuterated solvent taking into account the isotopic effects of the viscosity [44].

The self-diffusion coefficient D_0_ represents the translational movement of the solute at infinite dilution and, as shown by Equation (2), is dependent on the size and shape of the solute, temperature, and viscosity of the solvent. The dependence of the diffusion coefficient *D* versus solute concentration was plotted for all the PUs (Figure S12 in Supplementary Materials), being approximately linear for all polymers because the solutions are diluted. The self-diffusion coefficients *D*_0_ at infinite dilution (c = 0) are summarized in Table 3. The corresponding values of r_h_ obtained from Equation 2 are also presented in Table 3.

In the family of polyurethanes based on IPDI diisocyanate (**PU1**, **PU2**, and **PU3**), the hydrodynamic radius increases with *M*_n_ (see below in Section 3.5.2). When the IPDI is replaced by HMDI diisocyanate, as in the case of **PU4**, a substantial increase in the r_h_ is observed in comparison with polymers with similar molecular weights, such as **PU1** and **PU3**. The fragment derived from HMDI behaves like a larger (and more flexible) unit compared to IPDI, resulting in polymers of larger sizes for a similar molar ratio of the monomers used in the synthesis of the linear polyurethanes.

### 3.5. Determination of the Molecular Weights of the PUs

#### 3.5.1. Determination by GPC/SEC

The highest number-average molecular weight was obtained for **PU4**, the lowest one corresponds to **PU3** (Table 4), which are in agreement with the hydrodynamic radii of these polyurethanes. All the synthesized polyurethanes presented similar relatively narrow dispersities (*Đ* = *M*_w_/*M*_n_).

Since the GPC/SEC measurements (see chromatograms in Figure S13 in the Supplementary Materials) were calibrated with polystyrene (PS) standards, the values of *M*_n_ and *M*_w_ are relative to PS and may be quite different from the absolute values because the hydrodynamic volumes of the present PUs are considerably different from those of polystyrenes with equivalent molecular weights. 

#### 3.5.2. Determination by ^13^C NMR Spectroscopy

Quantitative ^1^H and ^13^C NMR are powerful tools to count monomer groups in homopolymers and copolymers of very well defined composition and narrow molecular weight distributions. The segmented linear polyurethanes herein described satisfy the above-mentioned criteria as they present well defined linear chains, low molecular weights, and dispersity indices below 1.5.

To improve the determination of *M*_n_ values of the present polyurethanes and attempt to obtain absolute values, the area of methyl Ca of PPG segments, centered at 17.2 ppm, which corresponds to an average of 17 carbons per PPG segment, was used as the reference instead of those of the copolymer end groups (see Tables S5–S8 of the Supplementary Materials for composition calculations). The polyurethanes average compositions obtained in this way are shown in Table 5 together with the calculated *M*_n_ values. These compositions agree with the monomer ratios used in the synthesis of polyurethanes **PU1**–**PU4** (see Table 2). 

The values obtained for *M*_n_ calculated by NMR are substantially lower than those determined by GPC/SEC (see Table 4), meaning that the hydrodynamic volumes of these PUs are considerably higher than those of the polystyrene standards with equivalent molar masses.

### 3.6. Thermal Properties of the PUs

**Figure 10:**
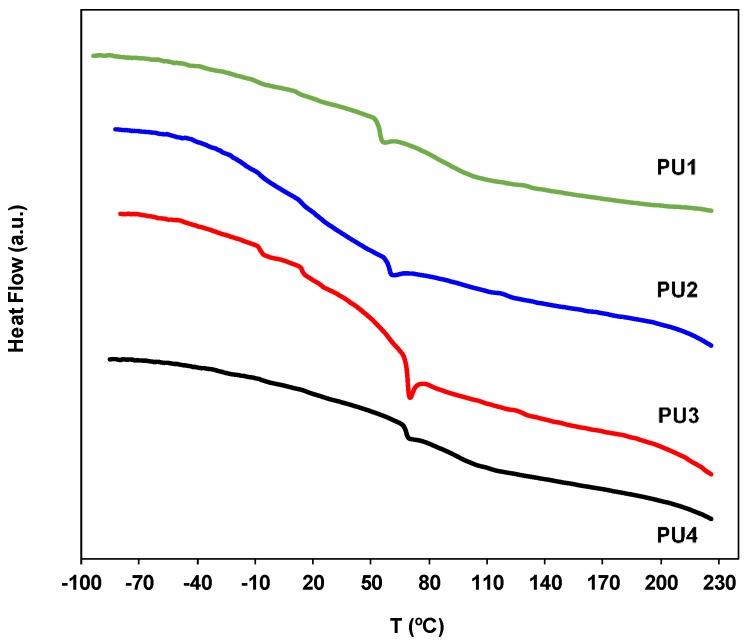
The DSC thermograms of the polyurethanes samples **PU1**–**PU4** (exo up).

The evaluation of the thermal behavior of the samples (polymers) is crucial for biomedical applications. For instance, if the value of the glass transition temperature (*T*_g_) of the polymer is higher than the human body temperature, the polymer is rigid (glass region). In contrast, if the *T*_g_ value is below the body temperature, the sample is in a rubber or elastomeric state. Figure 10 displays the DSC thermograms of the four materials under study in the range −100 to +230 °C. The DSC results presented refer to the second thermal cycle and the *T*_g_ value was determined as the onset point of the thermal event detected. All the samples are in the amorphous state and do not present phase separation. Thus, a unique glass transition above the human body temperature is detectable, at 54.6, 57.7, 69.0, and 68.0 °C, for the samples **PU1** to **PU4**, respectively, which indicate that these polyurethanes exhibit a lacquer/varnish behavior. No other thermal events were detected in addition to these glass transition temperatures, except in the case of **PU3**, which exhibits two additional small *T*_g_ events at lower temperatures (−7.2 and 14.4 °C), with the Δ*C*_p_ values ca. being three times smaller. The **PU3** sample has a higher content of low oligomers than the remaining samples, as can be observed in the corresponding GPC/SEC chromatograms (Figure S13 of the Supplementary Materials), which can possibly give rise to very small domains characterized by lower *T*_g_ values (likely corresponding to isolated short hard segments). 

### 3.7. Cytotoxicity Assay

**Figure 11:**
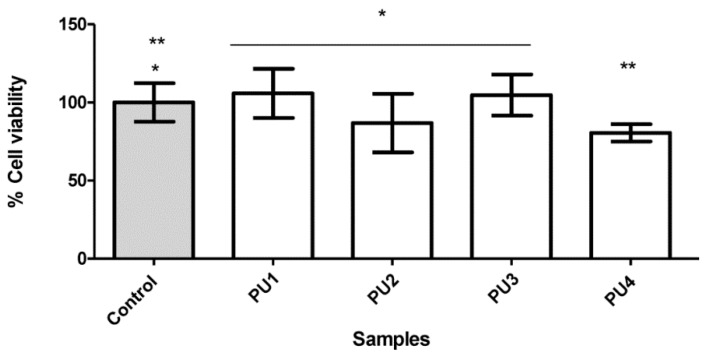
The HaCaT cell viability by MTT after proliferation under polyurethanes **PU1**–**PU4**. Control (glass slide). (mean ± SD) (*n* = 6). (* Not significantly different (*p* > 0.05), ** significantly different (*p* ˂ 0.05)).

The HaCaT keratinocyte cell line was selected to evaluate the cytotoxicity of the PUs synthesized in this work since these cells are the predominant cell type in the adult human epidermis, the outermost layer of the skin, thus, presenting an adequate in vitro cell model that better mimics in vivo conditions. The HaCaT cell viability, with 72 h of exposure to polyurethanes **PU1**–**PU4**, was evaluated by the MTT reduction assay [45] (Figure 11). The tests corresponding to **PU1**–**PU3** do not differ significantly from the control experiment (glass coverslip), whereas the cell viability for **PU4** is 80% ± 5% (i.e., significantly different from control for *p* < 0.05).

The cell viability was higher than 70% for all the PUs tested. Thus, these materials can be considered biocompatible according to this in vitro assay. These results are in accordance with the previously published results, where the PU nail lacquers were evaluated and found to be material biocompatible, being proposed for nail applications as safe pharmaceutical excipients [11].

## 4. Conclusions

A convenient synthesis for polyurethanes from renewable sources is described. The structure of these polyurethanes was determined and confirmed by FTIR, ^1^H, and ^13^C NMR spectroscopies of all four samples. The structures found confirm the higher reactivity of the secondary NCO group of IPDI compared to the primary group.

The quantitative ^13^C NMR spectroscopy analysis enabled the calculation of the polyurethanes compositions, number-average molecular weights, as well as of their diffusion coefficients (*D*) and hydrodynamic radii (*r*_h_) from the PGSE NMR experiments. Both values are in accordance with the molar ratio employed in the synthesis of the polyurethanes.

The polyurethanes were also characterized by GPC/SEC leading to molecular weights, which are considerably higher than the previous values since they are determined relative to the polystyrene standards, and relatively narrow molecular weight distributions, with dispersity indices *M*_w_/*M*_n_ < 1.5.

The DSC studies indicate that the four PU samples are in the amorphous state, being characterized by *T*_g_ values above the human body temperature.

The polyurethanes obtained were biocompatible with keratinocyte cells in the conditions used in the tests. This is a relevant property that must be taken into account when considering pharmaceutical excipients. For this reason, these newly synthesized polymers can be used in pharmaceutics, namely, as a component of nail lacquers for controlled-release drug delivery.

## Figures and Tables

**Table 1:** The molar ratios of the monomers employed in the synthesis of the quasi-prepolymer.

Monomers	Pre-1	Pre-2	Pre-3
IPDI	6	6	-
HMDI	-	-	6
PPG	1	2	1

**Table 2:** The molar and mass ratios of the monomers and prepolymer employed in the synthesis of the polyurethanes **PU1–PU4**, and the hard segment content (wt %).

Monomers	Polymers			
	PU1 n (mol)/m (g)	PU2 n (mol)/m (g)	PU3 n (mol)/m (g)	PU4 n (mol)/m (g)
IPDI	0.06/13.33	0.06/13.33	0.06/13.33	-
HMDI	-	-	-	0.06/15.74
PPG	0.01/10.00	0.02/20.00	0.01/10.00	0.01/10.00
D-isosorbide	0.05/7.30	0.05/7.30	0.06/8.76	0.05/7.30
Hard segment content (wt %) ^a^	67.4	50.8	68.8	69.7

^a^ Hard segment content (wt %) = (*m_isocyanate_* + *m_D-isosorbide_*)/(*m_isocyanate_* + *m_D-isosorbide_* + *m_PPG_*).

**Table 3:** The measured average values of *D*_0_ for the PUs determined by PGSE NMR and the corresponding hydrodynamic radii (*r*_h_) in DMSO-*d*_6_, at 30 °C.

Polyurethane	*D*_0_ × 10^11^ (m^2^/s^−1^)	*r_h_* (nm)
**PU1**	7.04	1.62 ± 0.04
**PU2**	4.49	2.65 ± 0.15
**PU3**	6.74	1.69 ± 0.04
**PU4**	4.51	2.52 ± 0.19

**Table 4:** The average molecular weights and dispersities determined by GPC/SEC ^a^.

Polyurethane No.	*M*_w_^a^ (Dalton)	*M*_n_^a^ (Dalton)	*Ð* (*M*_w_/*M*_n_)
**PU1**	15,600	10,300	1.50
**PU2**	19,200	12,800	1.48
**PU3**	9200	7200	1.27
**PU4**	23,500	15,100	1.55

^a^ In polystyrene units.

**Table 5:** The average compositions and *M*_n_ of **PU1**–**PU4** determined by NMR spectroscopy.

Polyurethane No.	Number of Isosorbide Units ^a^	Number of IPDI Units ^a^	Number of HDMI Units ^a^	Number of PPG Segments	*M_n_^b^* (Dalton)
**PU1**	5 (t)	6 (t)	-	1	3040
**PU2**	5 (t)	7 (t,b)	-	2	4270
**PU3**	6 (t)	6	-	1	3210
**PU4**	5 (t)	-	6 (t)	1	3380

^a^ t = at least one monomer is terminal; b = one monomer is in between PPG segments. ^b^ *M*_n_ values with uncertainties up to 15%.

## Supplementary Materials

The following are available online at http://www.mdpi.com/2073-4360/10/10/1170/s1.
