# Supplementary material for: Synthesis and Characterization of Isosorbide-Based Polyurethanes Exhibiting Low Cytotoxicity Towards HaCaT Human Skin Cells
Source: Polymers (Basel). 2018 Oct 20;10(10):1170. doi: 10.3390/polym10101170 (PMC6403884; doi:10.3390/polym10101170)
Supplement: Supplementary file 1 [file polymers-10-01170-s001.pdf]

Supplementary Material for

Synthesis and Characterization of Isosorbide-Based Polyurethanes Exhibiting Low Cytotoxicity Towards HaCaT Human Skin Cells

Barbara S. Gregorí Valdés[1,3], Clara S. B. Gomes[2], Pedro T. Gomes[2,*], José R. Ascenso[2,*], Hermínio P. Diogo[2], Lídia M. Gonçalves[3], Rui Galhano dos Santos[1], Helena M. Ribeiro[3], João C. Bordado[1,*]

[1] CERENA, Departamento de Engenharia Química, Instituto Superior Técnico, Universidade de Lisboa, Av. Rovisco Pais,1049-001 Lisboa, Portugal

[2] Centro de Química Estrutural, Departamento de Engenharia Química, Instituto Superior Técnico, Universidade de Lisboa, Av. Rovisco Pais, 1049-001 Lisboa, Portugal

[3] Research Institute for Medicine and Pharmaceutical Science (iMed.ULisboa), Faculty of Pharmacy, Universidade de Lisboa, Av. Prof. Gama Pinto, 1649-003 Lisboa, Portugal

E-mails:pedro.t.gomes@tecnico.ulisboa.pt, jose.ascenso@tecnico.ulisboa.pt, jcbordado@tecnico.ulisboa.pt

Atom numbering in chain fragments of polyurethanes used in NMR assignments

- Fragment with two monomers (Isosorbide and IPDI) of the chain of polyurethanes **PU1–PU3**:

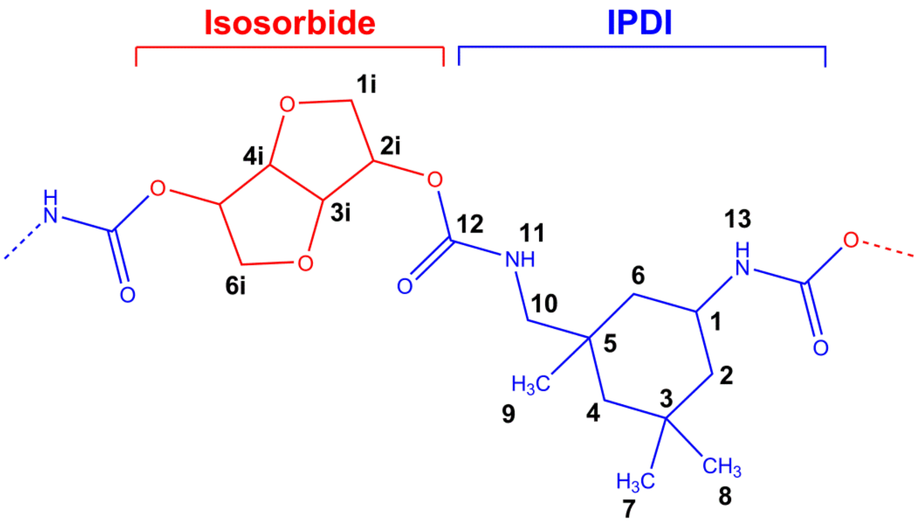

Fragment with two monomers (Isosorbide and HDMI) of the chain of polyurethane **PU4**:

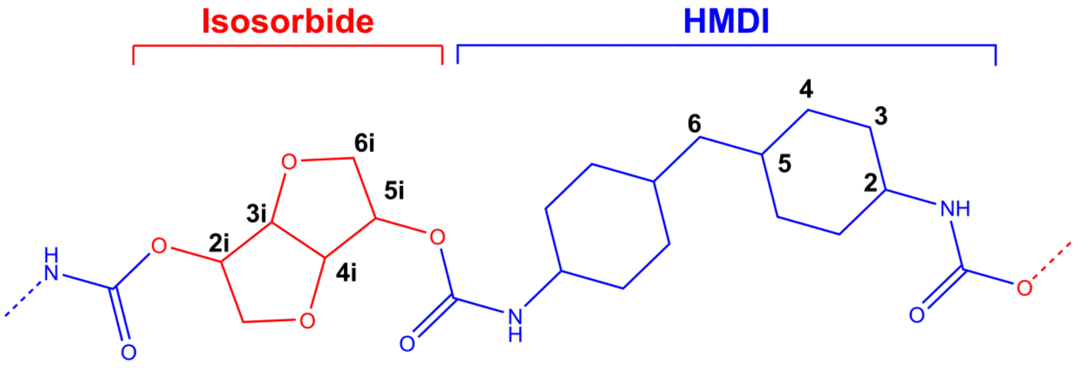

Average polypropyleneglycol (PPG) soft-blocks in **PU1–PU4**:

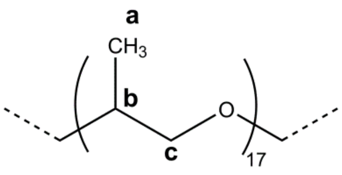

NMR data

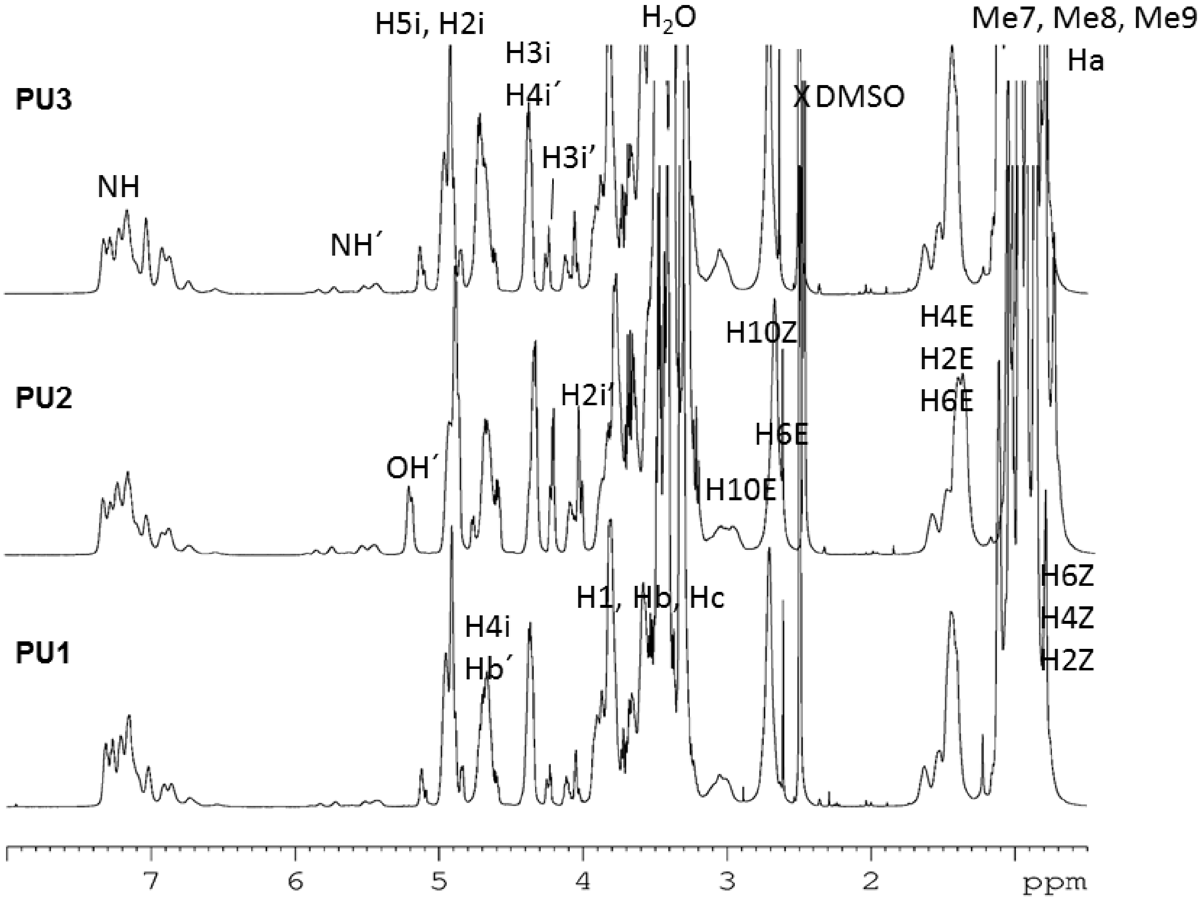

Figure S1. Assignment of the ^{1}H NMR spectra of polyurethanes **PU1**, **PU2** and **PU3** in DMSO-d_6. Protons of terminal groups are primed. Z and E refer to the *cis* and *trans* isomers of IDPI, respectively.

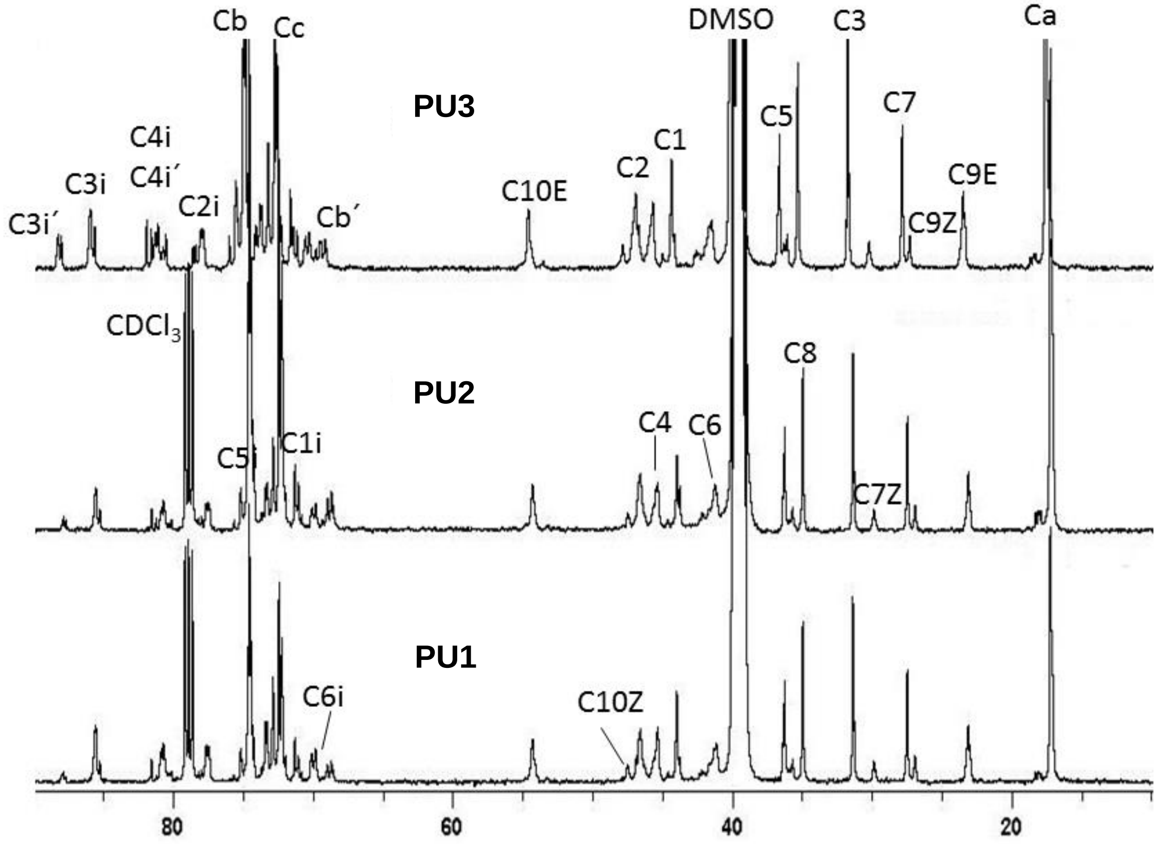

Figure S2. Assignment of the $^{13}C\{^{1}H\}$ NMR spectra of Isosorbide and IPDI repeating units of polyurethanes **PU1**, **PU2** and **PU3** in DMSO-d_6. Carbons of terminal groups are primed. Z and E refer to the *cis* and *trans* isomers of IDPI, respectively.

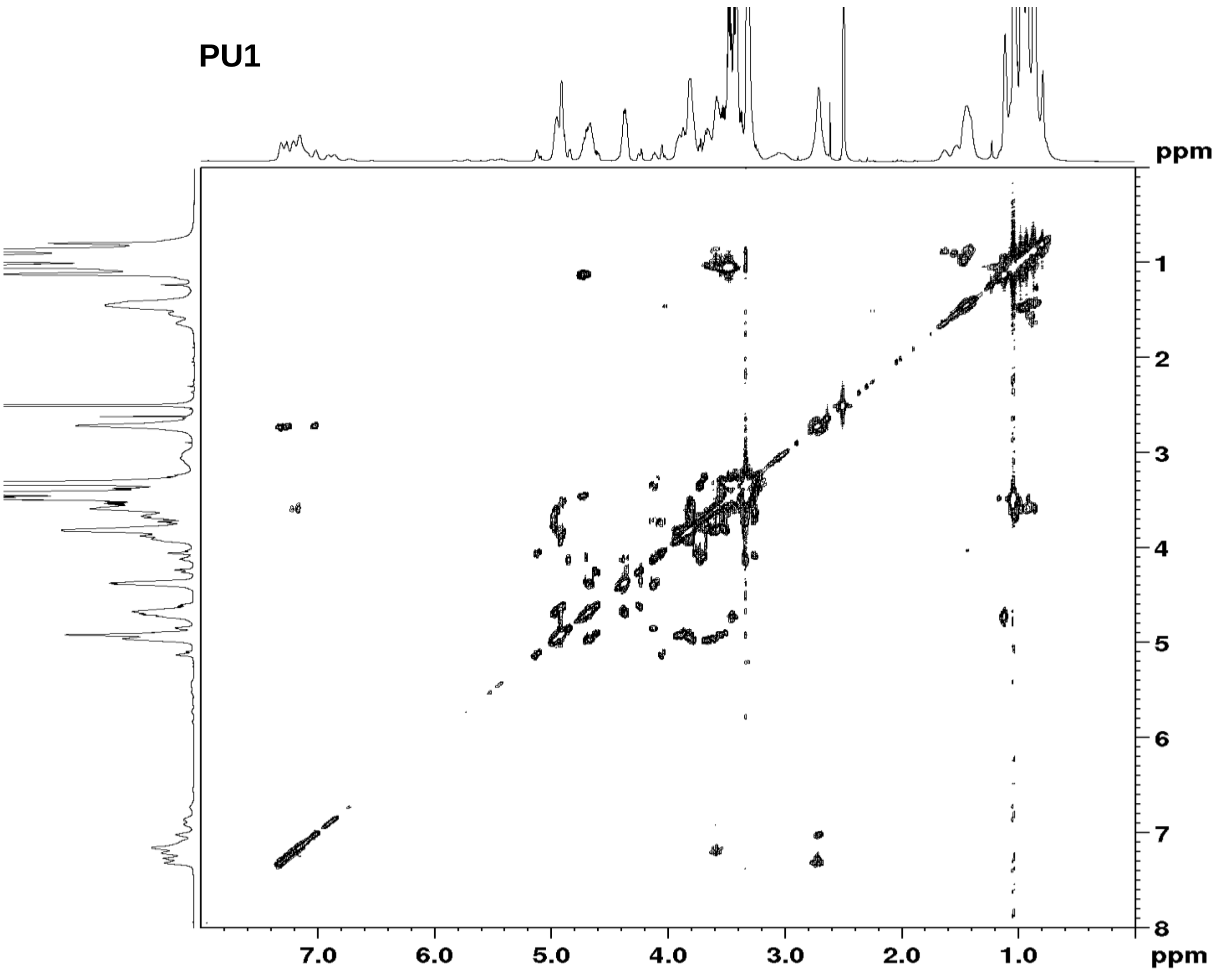

Figure S3. COSY spectrum of **PU1** in DMSO-d_6.

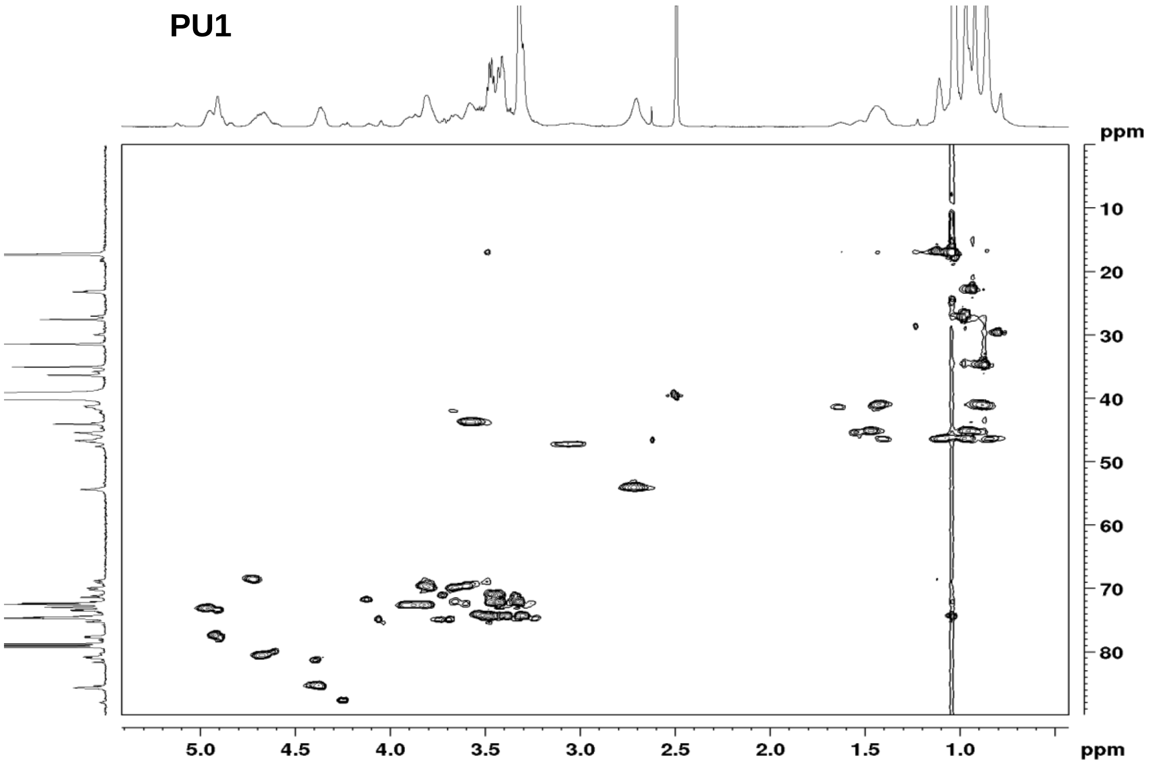

Figure S4. HSQC spectrum of **PU1** in DMSO-d_6.

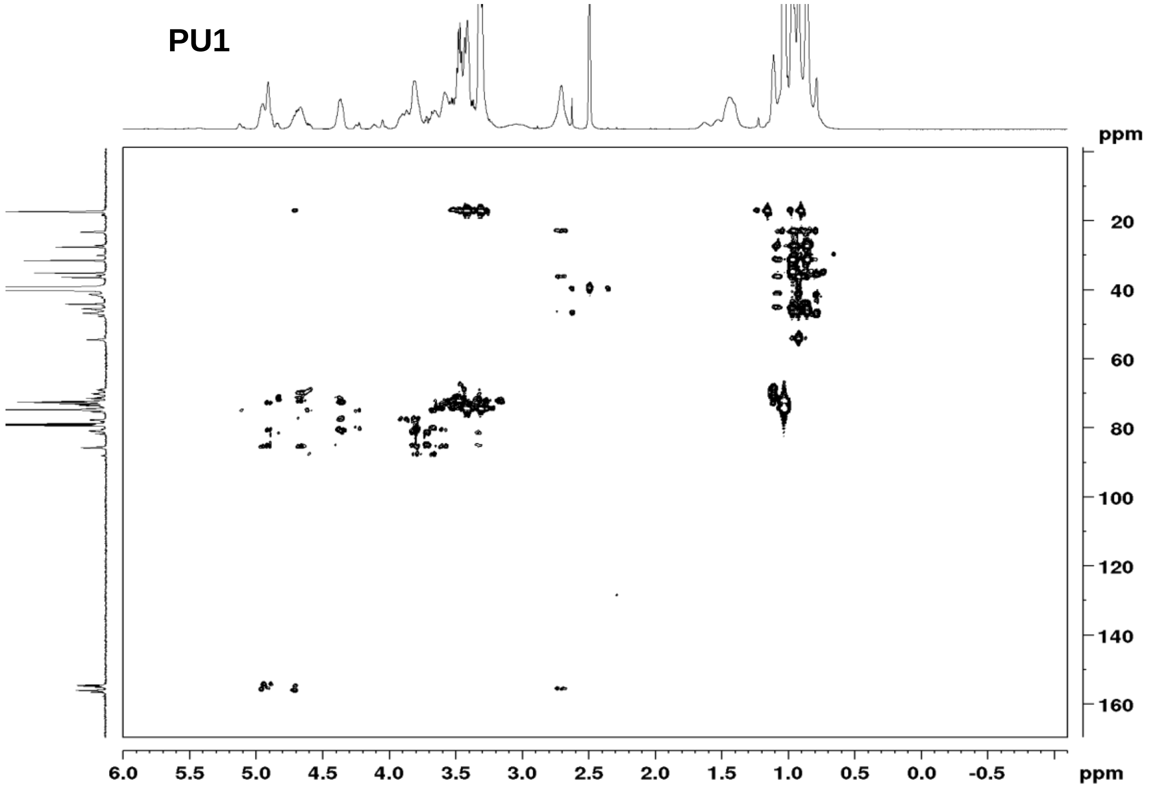

Figure S5. HMBC spectrum of **PU1** in DMSO-d_6.

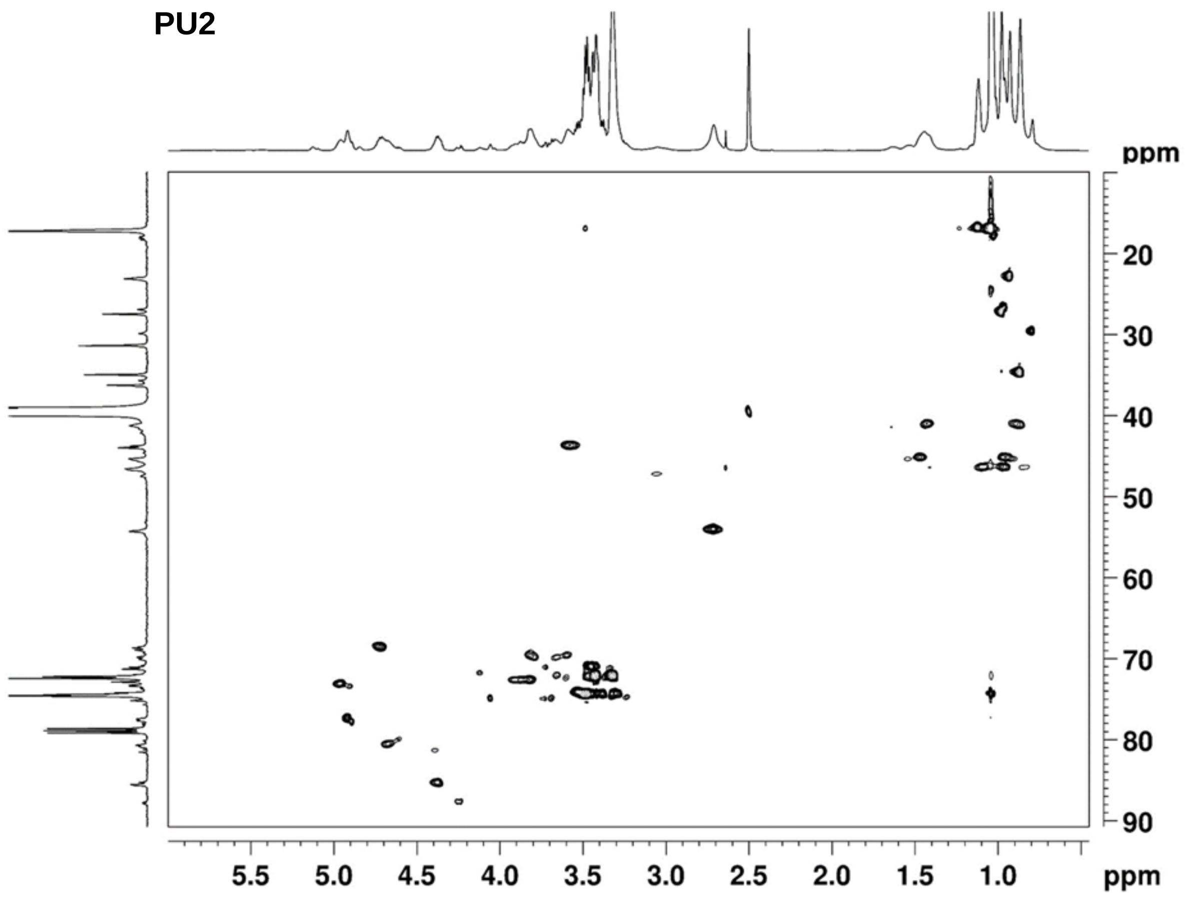

Figure S6. HSQC spectrum of **PU2** in DMSO-d_6.

PU3

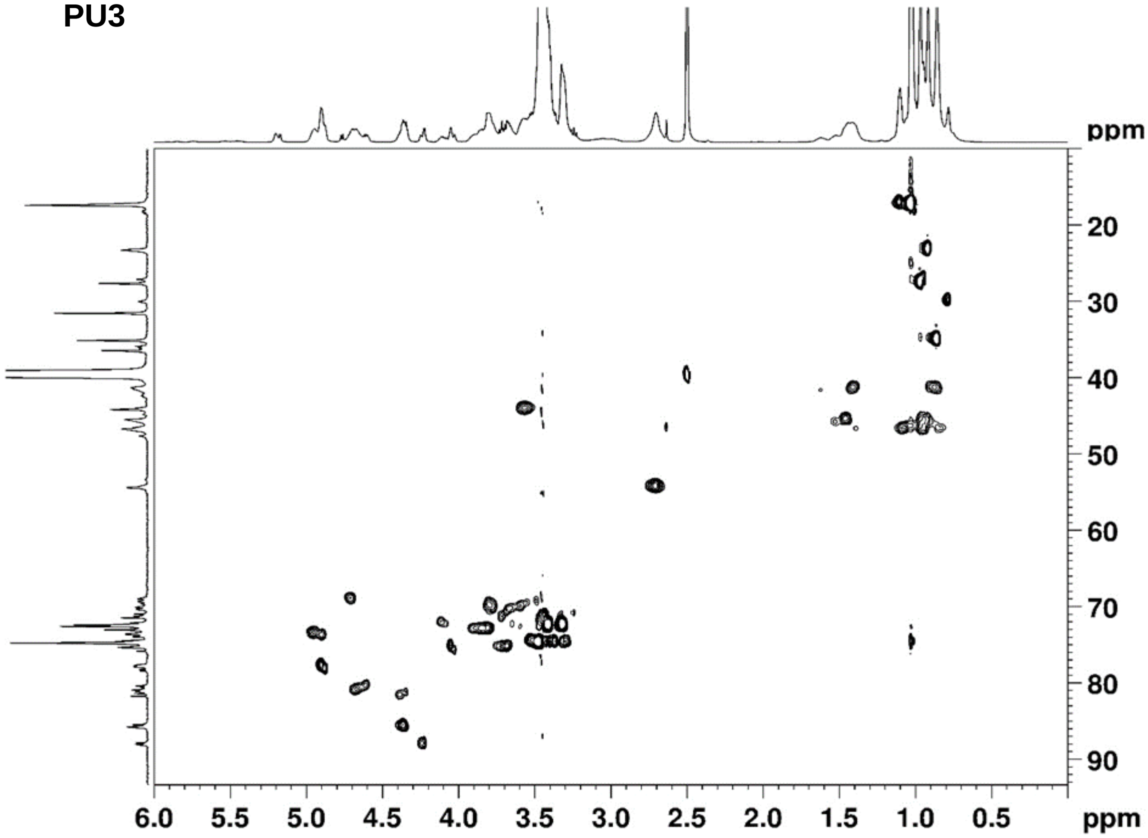

Figure S7. HSQC spectrum of **PU3** in DMSO-d_6.

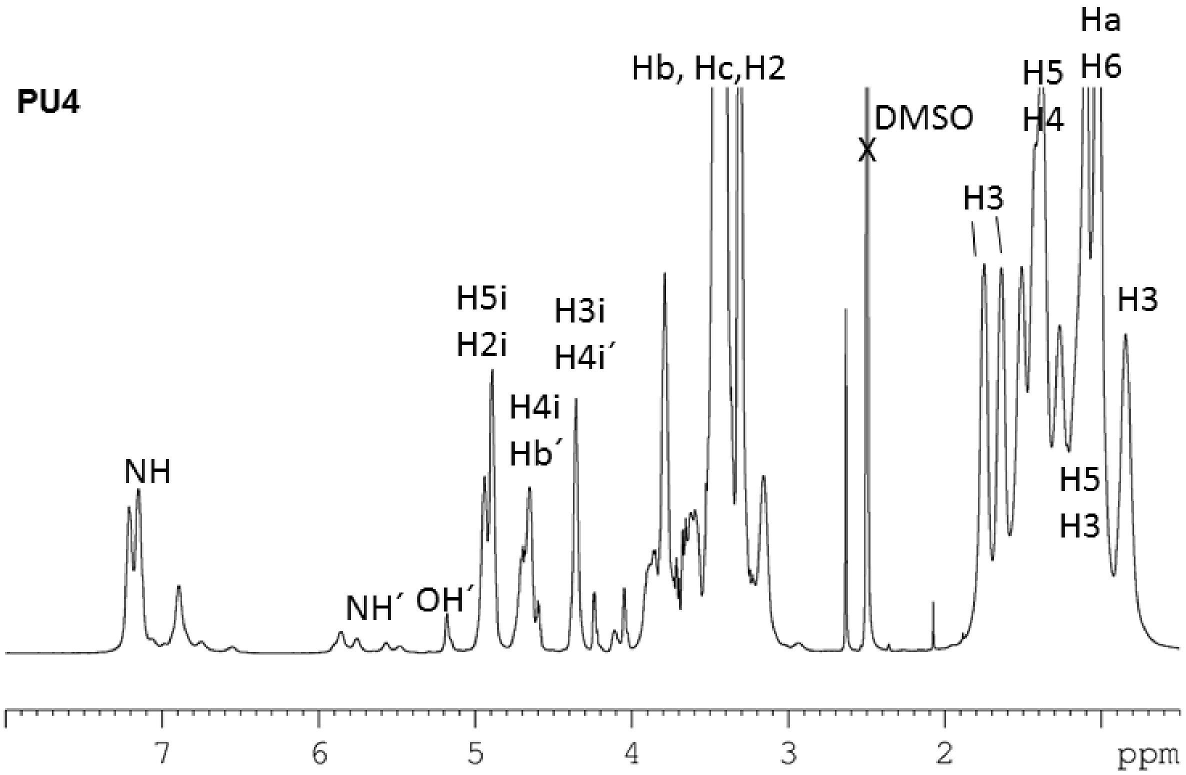

Figure S8. Assignment of the ^{1}H NMR spectrum of polyurethane **PU4** in DMSO-d_6. Protons of terminal groups are primed.

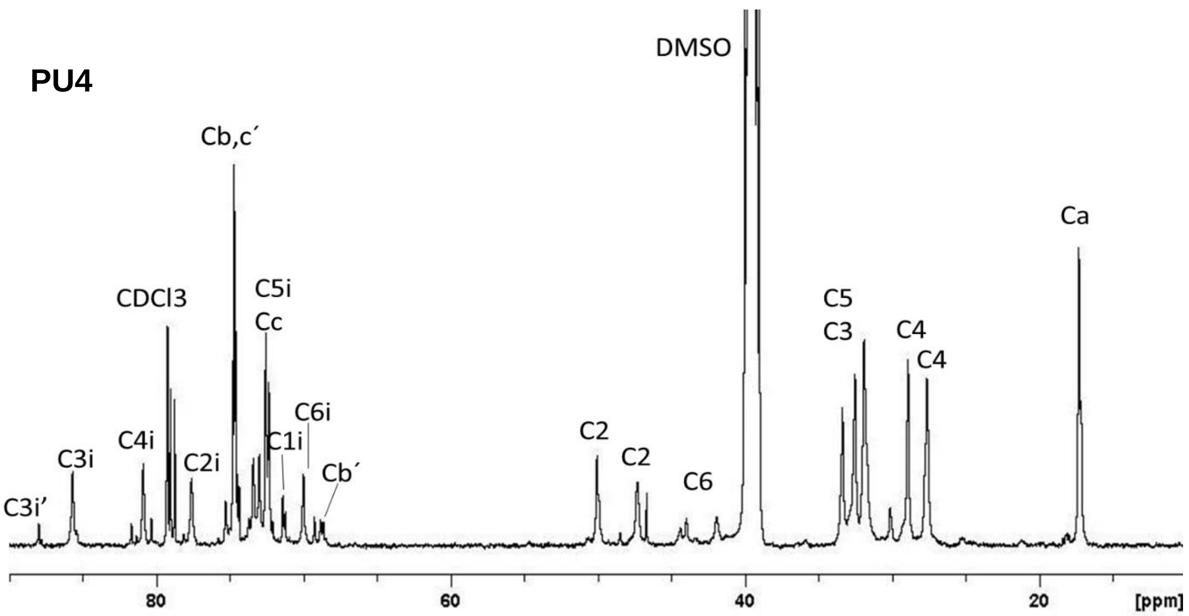

Figure S9. Assignment of the $^{13}C\{^{1}H\}$ NMR spectrum of Isosorbide and HMDI repeating units of polyurethane **PU4** in DMSO-d_6. Carbons of terminal groups are primed.

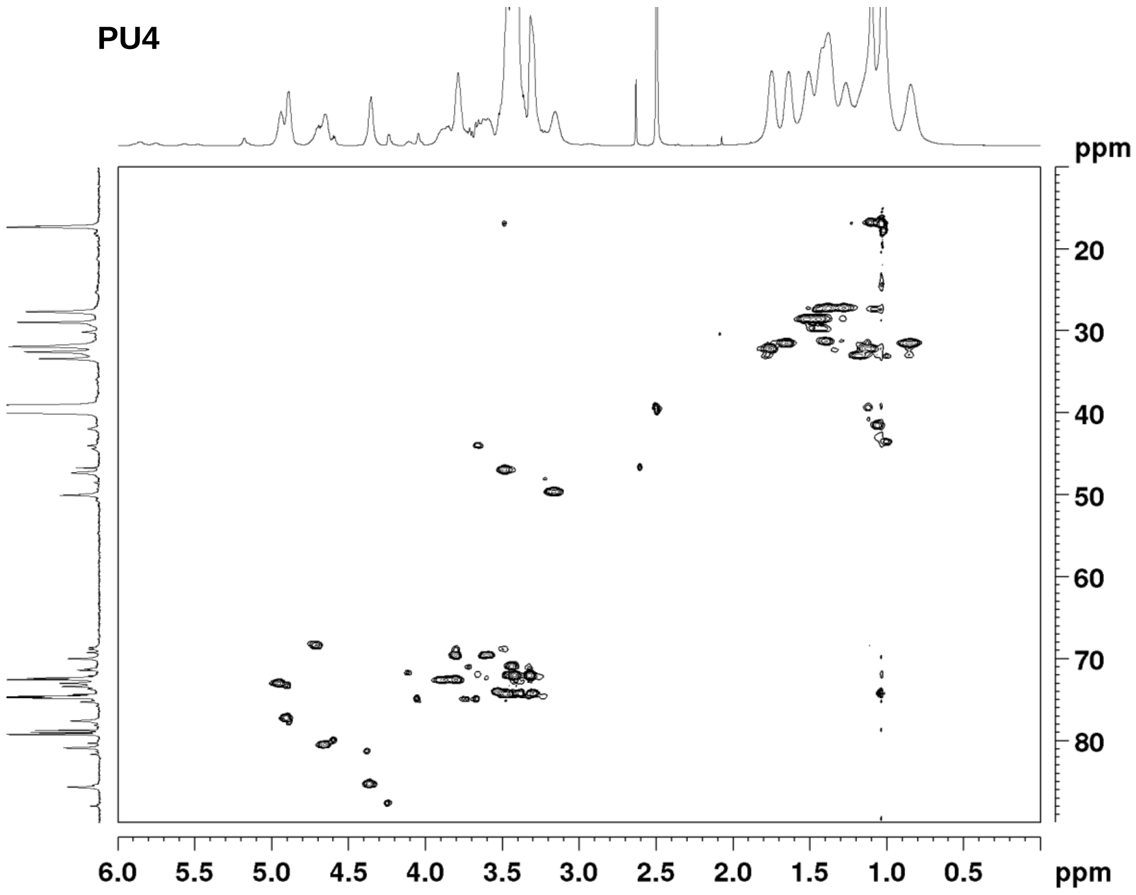

Figure S10. HSQC spectrum of **PU4** in DMSO-d_6.

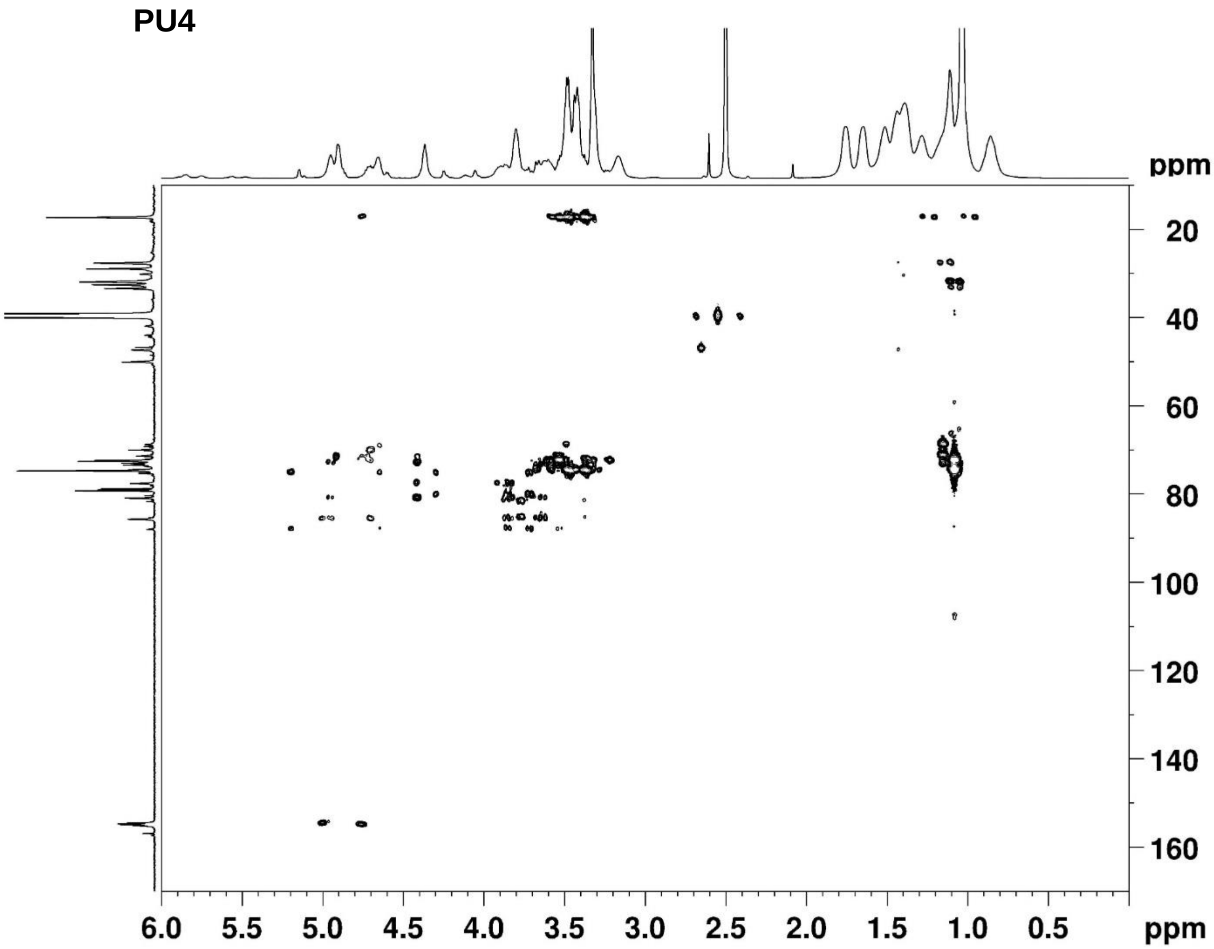

Figure S11. HMBC spectrum of **PU4** in DMSO-d_6.

Determination of self-diffusion coefficients D_0

The duration of the gradient pulses (δ) and the diffusion time (Δ) were optimized in order to obtain a residual signal of 2-5 % at the maximum gradient strength. The values used were 3.4 ms for the duration of the gradient pulses and 110 to 140 ms for the diffusion time. The gradient strength was incremented from 2% to 98% in a linear ramp with 16 steps. A delay of 15s between echoes was used. The gradients were previously calibrated using 99.9% pure D_2O as a standard. Each diffusion experiment produces a pseudo array of 16 spin echoes that were first FT processed in the t2 dimension using a LB of 0.2 Hz to generate a series of 1D spectra that were phased and baseline corrected prior to extraction of the diffusion coefficient by Gaussian fittings using the T_1/T_2 relaxation module of Topspin 3.1.

Table S1. Average diffusion coefficient D in relation to the concentration of **PU1**.

[**PU1**] (g/mL)	$D\times10^{11}$ (m^2/s^{-1})
3.42×10^{-4}	7.19
7.46×10^{-4}	7.25
1.74×10^{-3}	6.62
3.42×10^{-3}	6.61
1.14×10^{-2}	6.37

Table S2. Average diffusion coefficient D in relation to the concentration of **PU2**.

[**PU2**] (g/mL)	$D\times10^{11}$ (m^2/s^{-1})
3.66×10^{-4}	4.61
7.56×10^{-4}	4.17
1.62×10^{-3}	4.07
3.24×10^{-3}	4.04
1.22×10^{-2}	3.87

Table S3. Average diffusion coefficient D in relation to the concentration of **PU3**.

[**PU3**] (g/mL)	$D \times 10^{11}$ (m^2/s^{-1})
3.24×10^{-4}	6.62
7.56×10^{-4}	6.91
1.62×10^{-3}	6.61
3.24×10^{-3}	6.01
1.08×10^{-2}	6.01

Table S4. Average diffusion coefficient D in relation to the concentration of **PU4**.

[**PU4**] (g/mL)	$D \times 10^{11}$ (m^2/s^{-1})
2.94×10^{-4}	4.52
6.84×10^{-4}	4.08
1.47×10^{-3}	4.31
2.94×10^{-3}	4.62
9.80×10^{-3}	3.32

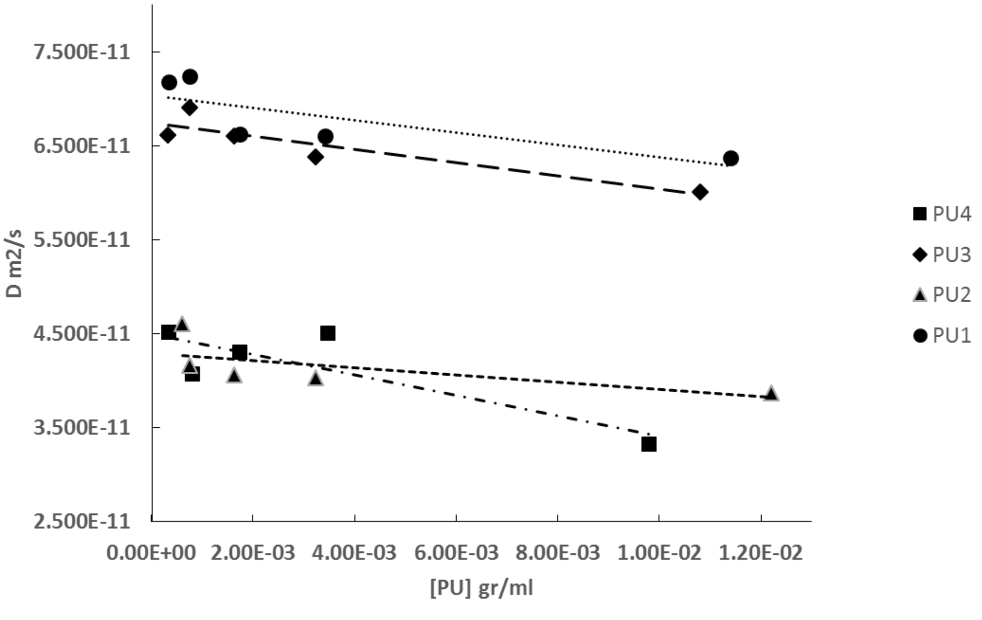

Figure S12. Plots of diffusion coefficients vs. concentrations of PUs.

GPC/SEC chromatograms

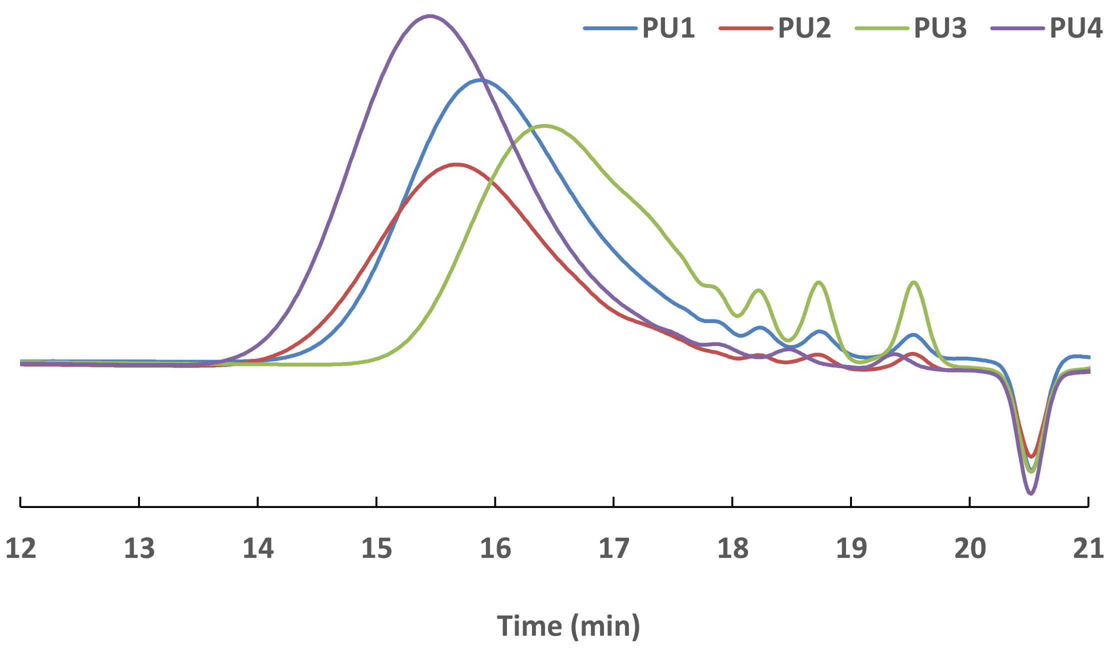

Figure S13. GPC/SEC chromatograms of **PU1-PU4**.

Average PUs compositions from ^{13}C NMR

Table S5. Average monomer composition of **PU1**.

Carbons	Relative area	No. carbons
CO	12.2	11 - 12 **carbonyls**
C3i´	0.5	1
C4i´	0.8	1 **terminal iosorbides**
C2i	4.2	4
C6i	4.1	4 **main chain isosorbides**
C4	5.8	6
C1	5.8	6
C8	6.5	6-7
C3	6.5	6-7
C7	6.3	6
C9	6.4	6 **isocyanates**
Cb	17.6	17 **PPG monomers**

Table S6. Average monomer composition of **PU2**.

**Carbons**	**Relative area**	**No. carbons**
CO	12.8	12-13 **carbonyls**
C3i´	1.9	2 **terminal isosorbides**
C3i	3.8	4
C4i	3.0	3
C2i	3.3	3
C6i	3.5	3 - 4 **main chain isosorbides**
C10	6.5	7
C2	8.2	8
C4	6.7	7
C1	6.7	7
C8	6.5	7
C3	6.7	7
C7	6.7	7
C9Z, C9E	6.6	6
Ca	34	34 **2 PPG blocks, 17 monomers each**

Table S7. Average monomer composition of **PU3**.

Carbons	Relative area	No. carbons
C3i´	1.9	2 **terminal isosorbides**
CO	10.8	11 **carbonyls**
C3i	3.9	4
C2i	4.0	4
Cb	17.1	17
C1i	4.1	4
C6i	3.8	4 **main chain isosorbides**
C4	5.4	5
C1	5.6	6.
C8	6.2	6
C3	6.4	6
C7	6.0	6
C9	6.2	6 **isocyanates**
Ca	17	17 **PPG monomers**

Table S8. Average monomer composition of **PU4**.

**Carbons**	**Relative area**	**No. Carbons**
CO	11.8	12 **carbonyls**
C3i′	0.6	1 **terminal isosorbides**
C3i	4.3	4
C4i,<br>C4i′	4.6	5
C2i	4.2	4
Cb	17.0	17
C6i	3.7	4 **main chain isosorbides**
C6	5.2	5
C2	5.3	5
C2	6.1	6
C4	13.6	14
C4	14.2	14 **6 isocyanates, one is terminal**
Ca	17.0	17 **PPG monomers**
